# Escalating vs Fixed Energy Defibrillation in Out-of-Hospital Cardiac Arrest Ventricular Fibrillation

**DOI:** 10.1001/jamanetworkopen.2025.7411

**Published:** 2025-04-29

**Authors:** Hanqi Tang, Ruoxue Wu, Lu Yin, Wenlin Hao, Jing Shi, Huadong Zhu, Shengyong Xu, Jun Xu

**Affiliations:** 1 Emergency Department, State Key Laboratory of Complex Severe and Rare Diseases, Peking Union Medical College Hospital, Chinese Academy of Medical Sciences and Peking Union Medical College, Beijing, China

## Abstract

**Importance:**

There is limited evidence on whether higher-energy defibrillation is preferred in patients experiencing out-of-hospital cardiac arrest (OHCA) with shockable rhythms.

**Objective:**

To investigate the optimal energy regimen for initial and subsequent defibrillation delivered by biphasic waveform automated external defibrillators (AEDs) in OHCA ventricular fibrillation (VF).

**Design, Setting, and Participants:**

This cohort study was conducted in 48 cities across China, from 2017 to 2023, among 342 patients with OHCA who experienced at least 1 shock.

**Exposures:**

Escalating higher-energy (200-300-360 J) defibrillation or fixed low-energy (200-200-200 J) defibrillation according to the AED program available for use.

**Main Outcome and Measures:**

Sustained and transient termination of VF and establishment of an organized rhythm after defibrillations were the main clinical outcomes.

**Results:**

A total of 342 patients with OHCA were included (mean [SD] age, 57.2 [20.6] years; 273 male [79.8%]) with 782 VF defibrillations; 218 patients (63.8%) with a total of 480 instances (61.4%) of VF rhythm received AED with escalating higher-energy regimens. Most VF episodes were effectively terminated transiently at the first shock (200 J in both groups) (94% in the escalating higher-energy group vs 93% in the fixed lower-energy group; *P* = .64), but only half remained terminated until the next rhythm analysis (49% vs 47%; *P* = .68). Comparatively, VF that received escalating higher-energy regimens were more likely to establish sustained organized rhythm (34% vs 25%; *P* = .008; absolute difference, 9% [95% CI, 2% to 16%]). In refractory VF rhythms, the percentage of cases where sustained organized rhythms were established was significantly greater in escalating higher-energy regimens after second shocks and above (24% vs 13%; *P* = .008; absolute difference, 11% [95% CI, 3% to 19%]) and third shocks and above (35% vs 18%; *P* = .003; absolute difference 17% [95% CI, 5% to 27%]).

**Conclusions and Relevance:**

In this retrospective cohort study of patients experiencing OHCA-VF, both the escalating higher-energy (200-300-360 J) regimen and the fixed low-energy (200-200-200 J) regimen were effective for transient VF termination at first shock, whereas the escalating higher-energy regimens were more likely to maintain termination and restore an organized rhythm. Higher-energy regimens were associated with better outcomes after all shocks, especially in patients with refractory VF.

## Key Points

**Question:**

Do patients with out-of-hospital cardiac arrest (OHCA) who receive shockable rhythms with higher defibrillation energy regimens have better prognosis?

**Findings:**

In this cohort study of 647 Chinese adults experiencing OHCA, both the escalating higher-energy (200-300-360 J) regimen and the fixed low-energy (200-200-200 J) regimen were effective for transient ventricular fibrillation (VF) termination at first shock, whereas the escalating higher-energy regimens were more likely to maintain VF termination and restore an organized rhythm. Higher-energy regimens were associated with better outcomes after all shocks, especially in patients with refractory VF.

**Meaning:**

These results suggest that escalating energy regimens might be preferred in patients with OHCA with shockable rhythms.

## Teaser

This cohort study of Chinese adults experiencing out-of-hospital cardiac arrest examines outcomes for terminating ventricular fibrillation using escalating higher-energy defibrillation vs a fixed low-energy regimen.

## Introduction

Cardiac arrest is one of the major public health issues of wide concern because of its high mortality and disability rates.^1^ With a large population of 1.4 million people, China has a crude incidence of emergency medical services (EMS)-assessed out-of-hospital cardiac arrest (OHCA) of 95.7 per 100 000 people reported,^2^ compared with 30.0 to 97.1 per 100 000 people across the world.^3^ The prognosis is poor, with only 1.2% of patients with OHCA surviving and 0.8% having favorable neurological outcomes at hospital discharge or 30 days in China,^2^ whereas 3.1% to 20.4% of patients with OHCA survive to hospital discharge across the world.^3^ It is estimated that ventricular fibrillation (VF) is the initial rhythm in up to 40% of patients of OHCA and that timely termination of VF contributes to 5% to 50% of survival.^4^ The chance of survival is 10 times greater for patients experiencing VF with rapid and effective defibrillation than for patients with asystole or pulseless electrical activity (PEA),^5,6^ and automatic external defibrillators (AEDs) play an essential role in what has been called the “chain of survival” in OHCA.

The importance of defibrillation in patients with OHCA-VF has been widely proven. However, the optimal defibrillation strategies are still under discussion, especially in refractory VF patients. The 2020 American Heart Association (AHA) Guidelines for Cardiopulmonary Resuscitation and Emergency Cardiovascular Care and European Resuscitation Council (ERC) Guidelines 2021 recommend that patients with failed defibrillation and recurrent fibrillation choose to increase the energy of defibrillation for defibrillation according to previous studies.^7,8,9^ The benefit of higher energy in defibrillation requires more evidence.

In this study, patients experiencing VF with out-of-hospital cardiac arrest who received defibrillation were retrospectively analyzed to investigate the association between defibrillation with different energy regimens and termination of VF episodes. The refractory VF population was analyzed separately and discussed.

## Method

### Study Design and Setting

This was a retrospective study conducted in patients experiencing OHCA who received AED defibrillations according to AHA protocols in 48 cities from 22 provinces across China from 2017 to 2023. The inclusion criteria were patients who experienced OHCA with AED provided by first responders or ambulance crews during prehospital resuscitation. Patients younger than 18 years of age, those who did not receive any shocks, or those with incomplete clinical medical records were excluded (eFigure 1 in Supplement 1).

AEDs used were of the same series models (Shenzhen Mindray Bio-Medical Electronics Co, Ltd), and the defibrillation regimens for each AED were programmed, determined before this study was performed, and remain unchanged since programmed. The AEDs provided either of 2 defibrillation regimens: escalating higher-energy (200-300-360 J) or fixed lower-energy (200-200-200 J) (eFigure 2 in Supplement 1). Defibrillation regimens for each patient were determined by AED available for use and were masked for all rescuers.

This study followed the Strengthening the Reporting of Observational Studies in Epidemiology (STROBE) reporting guideline. The study protocol was approved by the ethics committee of the Chinese Academy of Medical Science-Peking Union College Hospital. All data were deidentified and a waiver of informed consent was granted by the ethics committee.

### Patients and Data Collection

In this study, 342 patients who experienced VF among 647 patients with OHCA who received defibrillation were retrospectively analyzed to investigate the association between defibrillation with different energy regimens and termination of VF episodes. Demographic data, including age and sex, were recorded and anonymized by the researchers. Electrocardiogram (ECG) results are automatically recorded and analyzed by AEDs and subsequently extracted for analysis in this study. The ECGs are recorded continuously from the moment the defibrillator is powered on until it is powered off at the time of resuscitation termination or arrival at the hospital. No intervention for the routine management of patient care was included.

### Outcomes

Rhythms occurring after an electric shock include organized rhythms, ventricular quiescence (asystole), and VF and ventricular tachycardia (VT).^10^ All the ECG rhythms were automatically identified by the AED and proofread by at least 1 experienced clinician.

The primary outcome indicators were the resulting ECG rhythm, which was defined as the rhythm present at the next CPR interruption after defibrillation was delivered. The secondary outcome indicators were transient ECG rhythms present within 5 seconds after defibrillation was delivered. The outcomes of the defibrillation regimen are evaluated by percentages of conversion to organized rhythm and termination of VF episodes (including both asystole and conversion of organized rhythms). Organized rhythm is defined as the establishment of organized rhythm within 60 seconds. An organized rhythm required at least 2 QRS complexes separated by no more than 5 seconds. Refractory VF is defined as an initial presenting rhythm of VF or pulseless VT that was still present after 3 consecutive rhythm analyses and at least 2 standard defibrillations.^11^

### Data Analysis

Statistical analysis was performed with SPSS version 23.0 for Windows (SPSS, Inc). Categorical variables were compared via the χ^2^ test or Fisher exact test. Absolute differences were calculated between groups with a confidence level of 95%. Differences in results were considered statistically significant at *P* < .05 in 2-sided tests.

## Results

**Figure 1.**
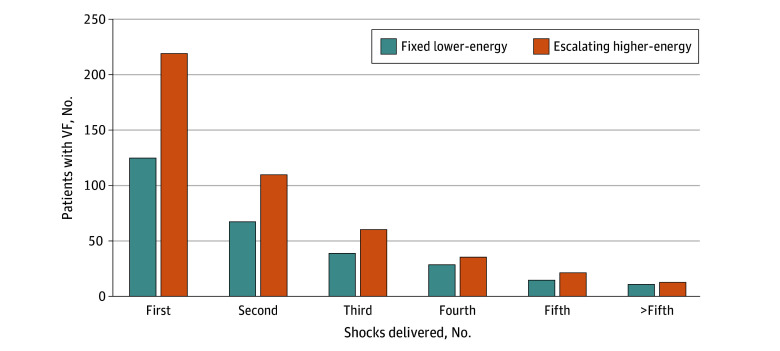
Patients in Each Ventricular Fibrillation (VF) Episode

Of 647 patients experiencing OHCA with connected AEDs, 342 patients (mean [SD] age, 57.2 [20.6] years; 273 male [79.8%]) whose initial ECG rhythm was VF and who received 782 defibrillations were enrolled for analysis. Among those patients, 218 (64%) received escalating higher-energy regimens, and 124 (36%) received fixed low-energy regimens (eFigure 1 in Supplement 1). The number of patients with VF decreased as the number of shocks increased in both groups (Figure 1). The mean (SD) age of the enrolled patients was 57.6 (20.6) years in escalating higher-energy group and 56.4 (20.8) years in fixed lower-energy group (*P* = .61). There were 173 male patients (79.4%) in escalating higher-energy group and 100 (80.6%) in fixed lower-energy group (*P* = .78). Initial ECG rhythms were mostly VF or VT (211 [96.8%] in escalating higher-energy group and 121 [97.6%] in the fixed lower-energy group; *P* = .68) (Table).

**Table.** Baseline Demographics of Patients With OHCA-VF

Variables	Patients, No. (%)		*P* value
	Fixed lower-energy (n = 124)	Escalating higher-energy (n = 218)	
Sex			
Female	24 (19.4)	45 (20.6)	
Male	100 (80.6)	173 (79.4)	.78
Age, mean (SD), y	56.4 (20.8)	57.6 (20.6)	.61
Initial rhythm			
VF and/or VT	121 (97.6)	211 (96.8)	.68
Asystole	1 (0.8)	2 (0.9)	.92
Other	2 (1.6)	5 (2.3)	.67

Abbreviations: OHCA, out-of-hospital cardiac arrest; VF, ventricular fibrillation; VT, ventricular tachycardia.

**Figure 2.**
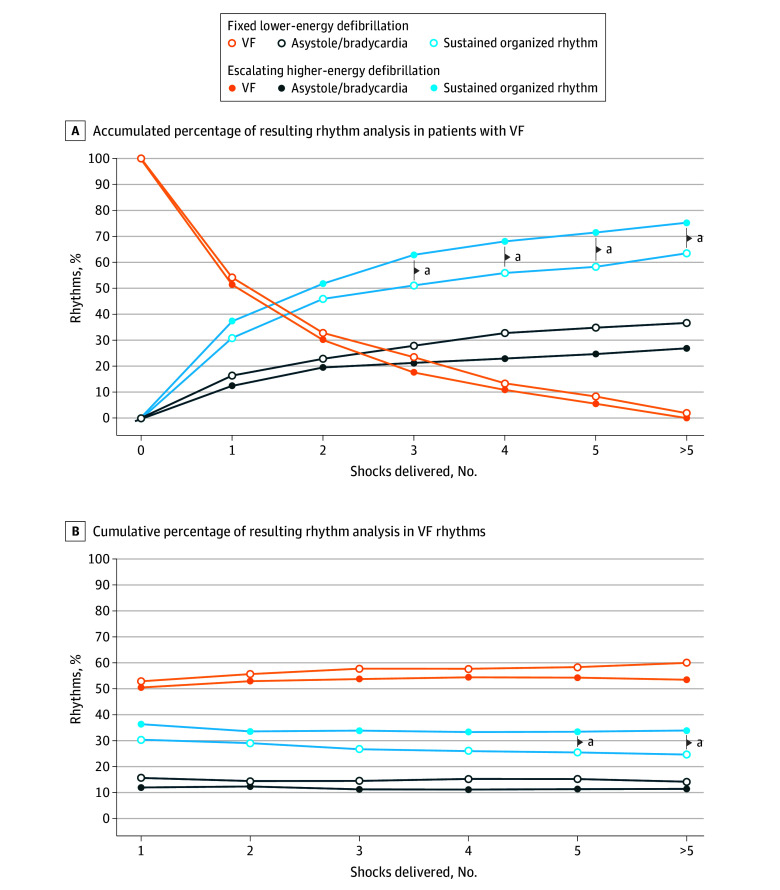
Comparison of Resulting Rhythms in Ventricular Fibrillation (VF) Between Fixed Lower-Energy and Escalating Higher-Energy Regimens ^a^*P* < .05.

For patients who received escalating higher-energy and fixed lower-energy AEDs, no significant difference was found between the first and third defibrillations when the resulting defibrillation rhythms were analyzed until the next CPR interruption (Figure 2A). When patients who received 3 or more shocks were included, significantly more organized rhythms (after the third shock, 132 of 218 [62%] vs 62 of 124 [50%]; *P* = .04; after the fourth shock, 142 of 218 [67%] vs 69 of 124 [55%]; *P* = .03; after the fifth shock, 149 of 218 [70%] vs 70 of 124 [57%]; *P* = .02; total, 157 of 218 [74%] vs 76 of 124 [62%]; *P* = .03) after shock delivery were observed in the escalating higher-energy group than in the fixed lower-energy group (Figure 2A; eTable 1 in Supplement 1). For VF episodes that received escalating higher-energy and fixed lower-energy regimens, the resulted ECG rhythm analysis revealed more VF conversion to organized rhythms in the escalating higher-energy group after 5 or more shocks were delivered (after fifth shock, 34% vs 26%; *P* = .03; total, 34% vs 25%; *P* = .008; absolute difference, 8.5% [95% CI, 2.4% to 15.6%) (Figure 2B). In each defibrillation episode, more VF episodes converted to organized rhythms after the third shock in the escalating higher-energy group (36% vs 16%; *P* = .04). (eFigure 3, eTables 1-3 in Supplement 1).

**Figure 3.**
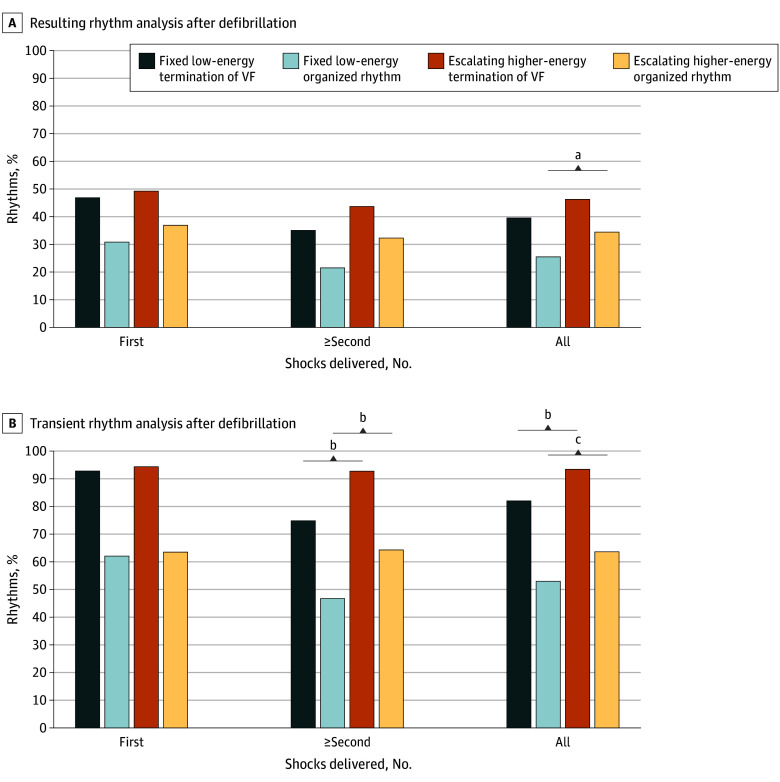
Comparison of the Resulting or Transient Rhythm Between the Fixed Lower-Energy and Escalating Higher-Energy Regimens ^a^*P* < .05. ^b^*P* < .001. ^c^*P* < .01.

All patients received 200 J defibrillations at the first shock in both groups, and no significant differences were observed in either the resulting rhythms (Figure 3A) or transient rhythms (Figure 3B). Both fixed lower-energy and escalating higher-energy AED regimens were highly effective in transient terminating VF episodes (115 of 124 [93%] vs 205 of 218 [94%]; *P* = .64) at the first shock, with comparable outcomes in establishing an organized rhythm (77 of 124 [62%] vs 138 of 218 [63%]; *P* = .82). VF episodes in the escalating higher-energy group received higher energy after the second shock and above (eFigure 2 in the Supplement), and rhythm analysis revealed that a significantly greater percentage of those patients terminated the VF (75% vs 93%; *P* < .001; absolute difference, 18.0% [95% CI, 11.3% to 24.7%]) and established an organized rhythm (47% vs 64%, *P* < .001; absolute difference, 17.5% [95% CI, 8.1% to 26.9%]) transiently (Figure 3B) (eTables 4-5 in Supplement 1).

**Figure 4.**
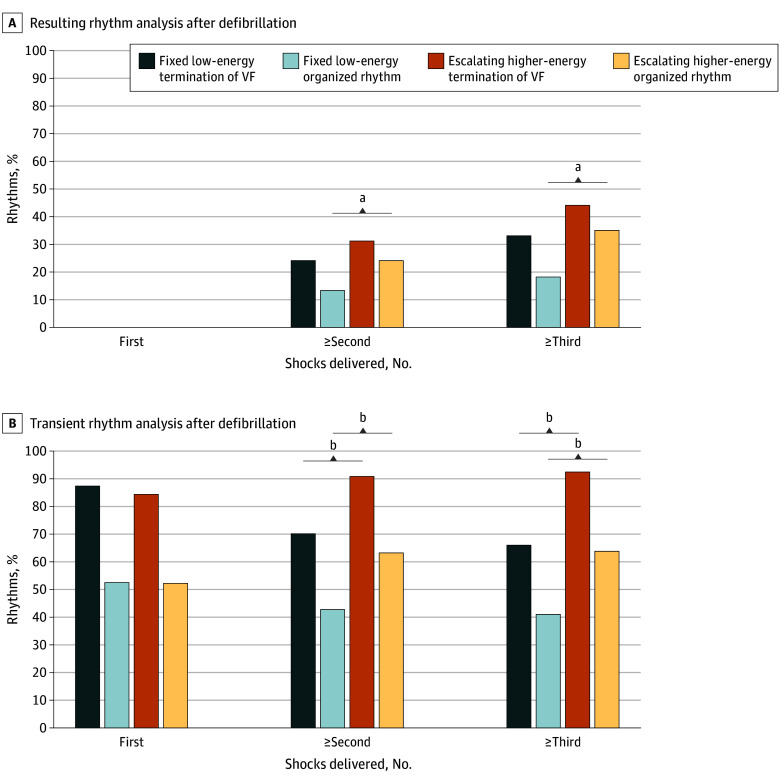
Comparison of the Resulting or Transient Rhythm Between Fixed Lower-Energy and Escalating Higher-Energy Regimens in Refractory Ventricular Fibrillation (VF) ^a^*P* < .05. ^b^*P* < .001.

In the refractory VF population, the resulting rhythm analysis revealed no significant difference in the termination VF percentage between groups, whereas the escalating higher-energy AED presented a significantly greater percentage of organized rhythm after the first shocks (200 J in both groups) were excluded (13% in the fixed lower-energy group vs 24% in the escalating higher-energy group; *P* = .008; absolute difference, 11.2% [95% CI, 2.9% to 19.4%]), and more VF episodes resulted in an organized rhythm after 360 J shocks in the escalating higher-energy group than after 200 J shocks in the fixed lower-energy group (35% vs 18%; *P* = .003; absolute difference, 17% [95% CI, 5.9% to 27.7%]) (Figure 4A). These patients also had an increased probability of terminating VF, although this result was not significant (escalating higher-energy group, 44% vs fixed low-energy group, 33%; *P* = .55).

Transient rhythm analysis (Figure 4B) revealed that higher-energy shocks were associated with a greater rate of termination of VF (second shock and above, 91% vs 70%; *P* < .001; absolute difference, 21% [95% CI, 12.9% to 28.7%; third shock or above, 93% vs 66%; *P* < .001; absolute difference, 26% [95% CI, 17.3% to 36.2%]) and the establishment of organized rhythms (second shock and above, 63% vs 43%; *P* < .001; absolute difference, 21% [95% CI, 10.3% to 31.0%]; third shock and above, 64% vs 41%; *P* < .001; absolute difference, 23% [95% CI, 10.8% to 35.1%]).

## Discussion

This retrospective study in patients experiencing OHCA-VF found that both the escalating higher-energy (200-300-360 J) regimen and the fixed low-energy (200-200-200 J) regimen were effective for transient VF termination at first shock, whereas escalating higher-energy regimens were more likely to maintain termination and restore an organized rhythm. Escalating higher-energy regimens were associated with better outcomes after all shocks, especially in patients with refractory VF.

The optimal AED energy regimens have been discussed for decades. Animal studies have shown that higher energy provides superior conversion rates compared with lower energy.^12,13,14,15,16^ In clinical patients experiencing OHCA-VF, a randomized, triple-masked study in 2005 revealed that escalating higher-energy regimens (200-300-360 J) are superior in patients with refractory VF, with a conversion rate of 36.6%, to fixed lower-energy regimens (150-150-150 J), with a conversion rate of 24.7% (*P* = .04).^9^ A higher-energy defibrillation regimen was reported to yield better outcomes in transient termination of VF, and patients receiving 360 J defibrillation had the highest cumulative conversion rate in another retrospective observational study.^17^ The present study shows similar results. Patients and rhythm analysis demonstrated that the escalating higher-energy group tended to fail conversion less frequently (Figure 2A) and presented a more organized rhythm after multiple shocks. The 2010 AHA guidelines and 2015 AHA guideline update guidelines recommend the first shock energy to follow the manufacturer protocol, while higher energy might be considered when multiple shocks are needed. In our study, the first shock in both groups was set as 200 J, and no differences were observed between the outcomes of patients after the first shock. The first shock at 200 J results in approximately 50% termination of VF and 31% to 37% sustained organized rhythm, with up to 93% to 94% transient conversion. The high efficiency of VF termination has been proven in AEDs from different manufacturers.^9,10,18,19,20^ In the BIPHSIC trial,^9^ patients randomly received shocks of 150 J or 200 J at the first shock, but no differences were observed. VF is effectively transiently terminated by low-energy defibrillation, whereas termination or organized rhythm postshock might be better maintained in those who receive higher-energy defibrillation.

Refractory VF is defined as an initial presenting rhythm of VF or pulseless VT that was still present after 3 consecutive rhythm analyses and at least 2 standard defibrillations in the present study. The definition of refractory VF is slightly different among studies.^11,21,22,23^ In the present study, patients with refractory VF rhythms who received third and subsequent shocks showed an increased probability of establishing an organized rhythm and terminating VF (although this second result was not significant). Moreover, the transient ECG rhythm postshock showed that the escalating higher-energy group had significantly better success rates at both the termination of VF and restored organized rhythms. These results are similar to those of previous clinical studies that demonstrated that increasing energy to 360 J improved the conversion rates for patients with difficult-to-defibrillate VF episodes.^24,25^ The present study found that up to 93% to 94% of patients terminated VF shortly after shock, but nearly half of those converted patients presented with VF again, which was defined as recurrent VF in some studies.^25,26^ Patients experiencing refractory VF are mostly not those patients resistant to defibrillation but are those patients who cannot maintain non-VF rhythms.

Traditionally, VF is categorized as turbulent cardiac electrical activity, which implies a large amount of irregularity in the electrical waves that underlie ventricular excitation.^27^ Clinical manifestations such as pulmonary edema and high body mass index^28^ might divert defibrillation current from the myocardium and are believed to be possible reasons for resistance to defibrillation or low-energy defibrillation at first shock, although the findings are not consistent among studies.^25^ However, our study revealed that most refractory VF episodes are shockable transiently but recurrently. The underlying causes of irregular cardiac electricity (such as cardiac coronary disease-induced cardiomyocyte ischemia) seem to play a more important role in those patients. One hypothesis from animal study suggests that arrhythmias initiated by focal mechanisms are insensitive to defibrillation due to ischemia myocardial with increased electrical thresholds.^29,30^ An AED with higher-energy regimens might be able to suppress abnormal electrical activity for a longer time, which allows more life-saving coronary perfusion. This hypothesis requires further animal and clinical studies.

### Limitations

Our study has several limitations. One of the main concerns of high-energy shock is myocardial injury, the data of which are not included in this study. The guidelines for cardiopulmonary resuscitation issued by the European Resuscitation Council state that studies in humans have not shown any harm (including biomarkers, ECG changes, and ejection fraction) for biphasic wave energies of up to 360 J.^31^ Second, the analysis included several factors, including the age and sex of the patients, number of shocks and number of VF episodes, but other important BLS data regarding rescuers, medications and prognoses were not available. This observational study can only demonstrate an association between different energy regimens and the success rate of defibrillation as opposed to a causal relationship. Third, the number of cases involved in the present study was limited. There are 2 main reasons: only about 30% of patients received resuscitation attempt in OHCA, and fewer with AED accessible. It was reported by Jiaqi Zhang et al^2^ that less than 0.1% of 35090 patients were assessed by bystanders with a public-access AED in a nationwide OHCA study in 17 months. Further prospective study with more OHCA cases is needed.

## Conclusions

In this retrospective cohort study of patients experiencing OHCA-VF in China, escalating higher-energy treatment (200-300-360 J) was associated with superior defibrillation results than the fixed low-energy group (200-200-200 J). Especially for the refractory VF population requiring multiple shocks, the escalating higher-energy regimen with 360 J was particularly advantageous. Therefore, when the first shock using conventional energy fails, defibrillation therapy using more energy is a better choice.

## Notes

Supplement 1. **eFigure 1.** Flowchart of Patient Enrollment **eFigure 2.** Illustrations of AED Regimens **eFigure 3.** Comparison of Resulting Rhythms in Ventricular Fibrillation (VF) Between Fixed Lower-Energy and Escalating Higher-Energy Regimens for Each Defibrillation **eTable 1.** Comparison of Percentage of Patients With Different Resulted ECG Rhythms Between Fixed Lower-Energy and Escalating Higher-Energy Defibrillations After Each Shock Delivered **eTable 2.** Comparison of Accumulative Percentage of Different Resulted ECG Rhythms Between Fixed Lower-Energy and Escalating Higher-Energy Defibrillations After Each Shock Delivered **eTable 3.** Comparison of Percentage of Different Resulted ECG Rhythms Between Fixed Lower-Energy and Escalating Higher-Energy Defibrillations for Each Shock Delivered **eTable 4.** Comparison of Accumulative Percentage of Different Transient ECG Rhythms Between Fixed Lower-Energy and Escalating Higher-Energy Defibrillations After Each Shock Delivered **eTable 5.** Comparison of Percentage of Different Transient ECG Rhythms Between Fixed Lower-Energy and Escalating Higher-Energy Defibrillations for Each Shock Delivered

Supplement 2.
Data Sharing Statement

## References

[1] Berg KM, Bray JE, Ng KC, ; Collaborators. 2023 International Consensus on Cardiopulmonary Resuscitation and Emergency Cardiovascular Care Science With Treatment Recommendations: summary from the basic life support; advanced life support; pediatric life support; neonatal life support; education, implementation, and teams; and first aid task forces. Circulation. 2023;148(24):e187-e280. doi:10.1161/CIR.0000000000001179 37942682 PMC10713008

[2] Zheng J, Lv C, Zheng W, ; BASIC-OHCA Coordinators and Investigators. Incidence, process of care, and outcomes of out-of-hospital cardiac arrest in China: a prospective study of the BASIC-OHCA registry. Lancet Public Health. 2023;8(12):e923-e932. doi:10.1016/S2468-2667(23)00173-1 37722403

[3] Kiguchi T, Okubo M, Nishiyama C, . Out-of-hospital cardiac arrest across the world: first report from the International Liaison Committee on Resuscitation (ILCOR). Resuscitation. 2020;152:39-49. doi:10.1016/j.resuscitation.2020.02.044 32272235

[4] Kleinman ME, Brennan EE, Goldberger ZD, . Part 5: adult basic life support and cardiopulmonary resuscitation quality: 2015 American Heart Association Guidelines update for cardiopulmonary resuscitation and emergency cardiovascular care. Circulation. 2015(18_suppl2):S414-S435. doi:10.1161/CIR.0000000000000259 26472993

[5] Pepe PE, Levine RL, Fromm RE Jr, Curka PA, Clark PS, Zachariah BS. Cardiac arrest presenting with rhythms other than ventricular fibrillation: contribution of resuscitative efforts toward total survivorship. Crit Care Med. 1993;21(12):1838-1843. doi:10.1097/00003246-199312000-00009 8252887

[6] Berdowski J, Berg RA, Tijssen JGP, Koster RW. Global incidences of out-of-hospital cardiac arrest and survival rates: systematic review of 67 prospective studies. Resuscitation. 2010;81(11):1479-1487. doi:10.1016/j.resuscitation.2010.08.006 20828914

[7] Panchal AR, Bartos JA, Cabañas JG, ; Adult Basic and Advanced Life Support Writing Group. Part 3: adult basic and advanced life support: 2020 American Heart Association Guidelines for Cardiopulmonary Resuscitation and Emergency Cardiovascular Care. Circulation. 2020;142(16_suppl_2)(suppl 2):S366-S468. doi:10.1161/CIR.0000000000000916 33081529

[8] Hess EP, Russell JK, Liu PY, White RD. A high peak current 150-J fixed-energy defibrillation protocol treats recurrent ventricular fibrillation (VF) as effectively as initial VF. Resuscitation. 2008;79(1):28-33. doi:10.1016/j.resuscitation.2008.04.028 18621462

[9] Stiell IG, Walker RG, Nesbitt LP, . BIPHASIC Trial: a randomized comparison of fixed lower versus escalating higher energy levels for defibrillation in out-of-hospital cardiac arrest. Circulation. 2007;115(12):1511-1517. doi:10.1161/CIRCULATIONAHA.106.648204 17353443

[10] Hess EP, Agarwal D, Myers LA, Atkinson EJ, White RD. Performance of a rectilinear biphasic waveform in defibrillation of presenting and recurrent ventricular fibrillation: a prospective multicenter study. Resuscitation. 2011;82(6):685-689. doi:10.1016/j.resuscitation.2011.02.008 21397382

[11] Cheskes S, Verbeek PR, Drennan IR, . Defibrillation strategies for refractory ventricular fibrillation. N Engl J Med. 2022;387(21):1947-1956. doi:10.1056/NEJMoa2207304 36342151

[12] Walker RG, Melnick SB, Chapman FW, Walcott GP, Schmitt PW, Ideker RE. Comparison of six clinically used external defibrillators in swine. Resuscitation. 2003;57(1):73-83. doi:10.1016/S0300-9572(02)00404-5 12668303

[13] Esibov A, Chapman FW, Melnick SB, Sullivan JL, Walcott GP. Minor variations in electrode pad placement impact defibrillation success. Prehosp Emerg Care. 2016;20(2):292-298. doi:10.3109/10903127.2015.1076095 26383036

[14] Ristagno G, Yu T, Quan W, Freeman G, Li Y. Current is better than energy as predictor of success for biphasic defibrillatory shocks in a porcine model of ventricular fibrillation. Resuscitation. 2013;84(5):678-683. doi:10.1016/j.resuscitation.2012.09.029 23032689

[15] Li Y, Ristagno G, Yu T, Bisera J, Weil MH, Tang W. A comparison of defibrillation efficacy between different impedance compensation techniques in high impedance porcine model. Resuscitation. 2009;80(11):1312-1317. doi:10.1016/j.resuscitation.2009.08.004 19720442

[16] Chen B, Yu T, Ristagno G, Quan W, Li Y. Average current is better than peak current as therapeutic dosage for biphasic waveforms in a ventricular fibrillation pig model of cardiac arrest. Resuscitation. 2014;85(10):1399-1404. doi:10.1016/j.resuscitation.2014.06.029 25010783

[17] Baker PW, Conway J, Cotton C, ; Clinical Investigators. Defibrillation or cardiopulmonary resuscitation first for patients with out-of-hospital cardiac arrests found by paramedics to be in ventricular fibrillation? A randomised control trial. Resuscitation. 2008;79(3):424-431. doi:10.1016/j.resuscitation.2008.07.017 18986748

[18] Soar J, Böttiger BW, Carli P, . European Resuscitation Council Guidelines 2021: adult advanced life support. Resuscitation. 2021;161:115-151. doi:10.1016/j.resuscitation.2021.02.010 33773825

[19] Poole JE, White RD, Kanz KG, ; LIFE Investigators. Low-energy impedance-compensating biphasic waveforms terminate ventricular fibrillation at high rates in victims of out-of-hospital cardiac arrest. J Cardiovasc Electrophysiol. 1997;8(12):1373-1385. doi:10.1111/j.1540-8167.1997.tb01034.x 9436775

[20] Stothert JC, Hatcher TS, Gupton CL, Love JE, Brewer JE. Rectilinear biphasic waveform defibrillation of out-of-hospital cardiac arrest. Prehosp Emerg Care. 2004;8(4):388-392. doi:10.1080/312704000760 15625999

[21] Nichol G, Sayre MR, Guerra F, Poole J. Defibrillation for ventricular fibrillation: a shocking update. J Am Coll Cardiol. 2017;70(12):1496-1509. doi:10.1016/j.jacc.2017.07.778 28911514

[22] Coult J, Yang BY, Kwok H, . Prediction of shock-refractory ventricular fibrillation during resuscitation of out-of-hospital cardiac arrest. Circulation. 2023;148(4):327-335. doi:10.1161/CIRCULATIONAHA.122.063651 37264936 PMC13249127

[23] Cheskes S, Drennan IR, Turner L, Pandit SV, Dorian P. The impact of alternate defibrillation strategies on shock-refractory and recurrent ventricular fibrillation: a secondary analysis of the DOSE VF cluster randomized controlled trial. Resuscitation. 2024;198:110186. doi:10.1016/j.resuscitation.2024.110186 38522736

[24] Walker RG, Koster RW, Sun C, . Defibrillation probability and impedance change between shocks during resuscitation from out-of-hospital cardiac arrest. Resuscitation. 2009;80(7):773-777. doi:10.1016/j.resuscitation.2009.04.002 19423211

[25] Koster RW, Walker RG, Chapman FW. Recurrent ventricular fibrillation during advanced life support care of patients with prehospital cardiac arrest. Resuscitation. 2008;78(3):252-257. doi:10.1016/j.resuscitation.2008.03.231 18556106

[26] Telesz BJ, Hess EP, Atkinson E, White RD. Recurrent ventricular fibrillation: experience with first responders prior to advanced life support interventions. Resuscitation. 2015;88:138-142. doi:10.1016/j.resuscitation.2014.10.010 25447428

[27] Jalife J. Ventricular fibrillation: mechanisms of initiation and maintenance. Annu Rev Physiol. 2000;62:25-50. doi:10.1146/annurev.physiol.62.1.25 10845083

[28] Ogunnaike BO, Whitten CW, Minhajuddin A, ; American Heart Association’s Get With The Guidelines-Resuscitation Investigators. Body mass index and outcomes of in-hospital ventricular tachycardia and ventricular fibrillation arrest. Resuscitation. 2016;105:156-160. doi:10.1016/j.resuscitation.2016.05.028 27290990

[29] Walcott GP, Killingsworth CR, Smith WM, Ideker RE. Biphasic waveform external defibrillation thresholds for spontaneous ventricular fibrillation secondary to acute ischemia. J Am Coll Cardiol. 2002;39(2):359-365. doi:10.1016/S0735-1097(01)01723-5 11788232

[30] Qin H, Walcott GP, Killingsworth CR, Rollins DL, Smith WM, Ideker RE. Impact of myocardial ischemia and reperfusion on ventricular defibrillation patterns, energy requirements, and detection of recovery. Circulation. 2002;105(21):2537-2542. doi:10.1161/01.CIR.0000016702.86180.F6 12034662

[31] Deakin CD, Nolan JP; European Resuscitation Council. European Resuscitation Council guidelines for resuscitation 2005. Section 3. Electrical therapies: automated external defibrillators, defibrillation, cardioversion and pacing. Resuscitation. 2005;67(suppl 1):S25-S37. doi:10.1016/j.resuscitation.2005.10.008 16321714

