# Error in Figure 4

**DOI:** 10.1001/jamanetworkopen.2025.51210

**Published:** 2025-12-08

In the Original Investigation titled “Escalating vs Fixed Energy Defibrillation in Out-of-Hospital Cardiac Arrest Ventricular Fibrillation,”^1^ published April 29, 2025, an incorrect axis label was used in Panels A and B of Figure 4. The label “All” should have read “≥Third.” This article has been corrected.^1^

## References

[1] Tang H, Wu R, Yin L, . Escalating vs fixed energy defibrillation in out-of-hospital cardiac arrest ventricular fibrillation. JAMA Netw Open. 2025;8(4):e257411. doi:10.1001/jamanetworkopen.2025.7411 40299385 PMC12042058

