# Supplementary material for: Escalating vs Fixed Energy Defibrillation in Out-of-Hospital Cardiac Arrest Ventricular Fibrillation
Source: JAMA Netw Open. 2025 Apr 29;8(4):e257411. doi: 10.1001/jamanetworkopen.2025.7411 (PMC12042058; doi:10.1001/jamanetworkopen.2025.7411)
Supplement: Supplement 1. — eFigure 1. Flowchart of Patient Enrollment eFigure 2. Illustrations of AED Regimens eFigure 3. Comparison of Resulting Rhythms in Ventricular Fibrillation (VF) Between Fixed Lower-Energy and Escalating Higher-Energy Regimens for Each Defibrillation eTable 1. Comparison of Percentage of Patients With Different Resulted ECG Rhythms Between Fixed Lower-Energy and Escalating Higher-Energy Defibrillations After Each Shock Delivered eTable 2. Comparison of Accumulative Percentage of Different Resulted ECG Rhythms Between Fixed Lower-Energy and Escalating Higher-Energy Defibrillations After Each Shock Delivered eTable 3. Comparison of Percentage of Different Resulted ECG Rhythms Between Fixed Lower-Energy and Escalating Higher-Energy Defibrillations for Each Shock Delivered eTable 4. Comparison of Accumulative Percentage of Different Transient ECG Rhythms Between Fixed Lower-Energy and Escalating Higher-Energy Defibrillations After Each Shock Delivered eTable 5. Comparison of Percentage of Different Transient ECG Rhythms Between Fixed Lower-Energy and Escalating Higher-Energy Defibrillations for Each Shock Delivered [file jamanetwopen-e257411-s001.pdf]

Supplemental Online Content

Tang H, Wu R, Yin L, et al. Escalating vs fixed energy defibrillation in out-of-hospital cardiac arrest ventricular fibrillation. *JAMA Netw Open.* 2025;8(4):e257411. doi:10.1001/jamanetworkopen.2025.7411

eFigure 1. Flowchart of Patient Enrollment

eFigure 2. Illustrations of AED Regimens

eFigure 3. Comparison of Resulting Rhythms in Ventricular Fibrillation (VF) Between Fixed Lower-Energy and Escalating Higher-Energy Regimens for Each Defibrillation

eTable 1. Comparison of Percentage of Patients With Different Resulted ECG Rhythms Between Fixed Lower-Energy and Escalating Higher-Energy Defibrillations After Each Shock Delivered

eTable 2. Comparison of Accumulative Percentage of Different Resulted ECG Rhythms Between Fixed Lower-Energy and Escalating Higher-Energy Defibrillations After Each Shock Delivered

eTable 3. Comparison of Percentage of Different Resulted ECG Rhythms Between Fixed Lower-Energy and Escalating Higher-Energy Defibrillations for Each Shock Delivered

eTable 4. Comparison of Accumulative Percentage of Different Transient ECG Rhythms Between Fixed Lower-Energy and Escalating Higher-Energy Defibrillations After Each Shock Delivered

eTable 5. Comparison of Percentage of Different Transient ECG Rhythms Between Fixed Lower-Energy and Escalating Higher-Energy Defibrillations for Each Shock Delivered

This supplemental material has been provided by the authors to give readers additional information about their work.

eFigure 1 Flowchart of patient enrollment. In this observational study, 342 out-of-hospital cardiac arrest patients were enrolled, with 124 receiving fixed-lower-energy defibrillation and 218 receiving escalating-higher-energy defibrillation.

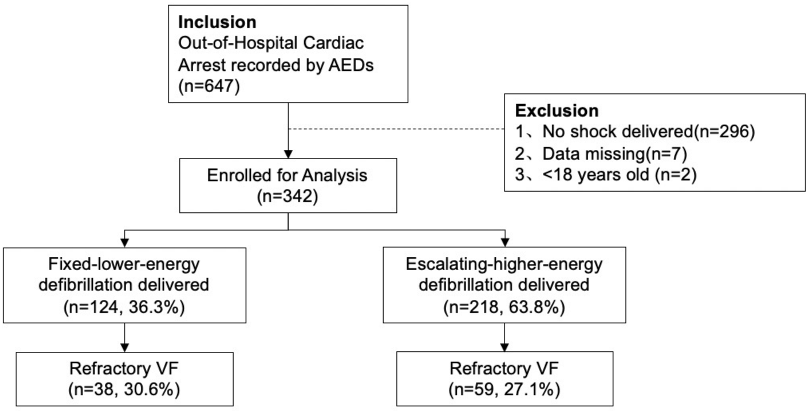

© 2025 Tang H et al. *JAMA Network Open.*

eFigure 2. Illustrations of AED regimens. The AEDs provided either of two defibrillation regimens: escalating-higher-energy (200--300--360J) and fixed-lower-energy (200--200--200J). CPR=cardiopulmonary resuscitation.

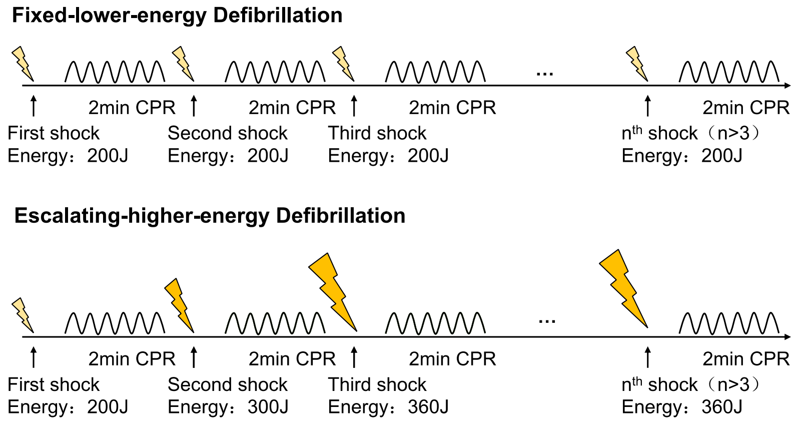

© 2025 Tang H et al. *JAMA Network Open.*

eFigure 3 Comparison of resulting rhythms in ventricular fibrillation (VF) between fixed-lower-energy and escalating-higher-energy regimens for each defibrillation. The green and blue bars refer to the fixed-lower-energy and escalating-higher-energy regimens, respectively. The light, middle, and dark colors refer to organized rhythm, asystole/bradycardia, and VF, respectively. *=p<0.05.

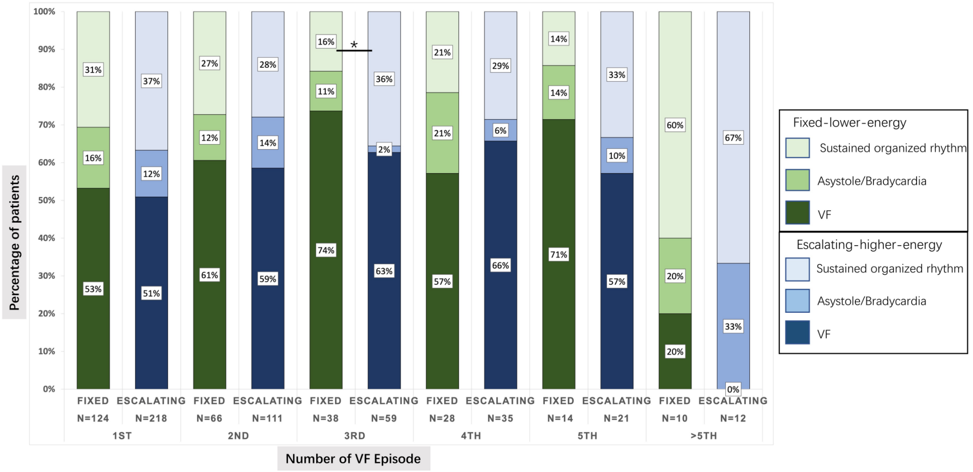

© 2025 Tang H et al. *JAMA Network Open.*

eTable 1. Comparison of percentage of patients with different resulted ECG rhythms between fixed-lower-energy and escalating-higher-energy defibrillations after each shock delivered

Number of Shock Delivered		Fixed-Lower-Energy Defibrillation n (%)	Escalating-Higher-Energy Defibrillation n (%)	p-value*
**1**	VF	66 (53%)	111 (51%)	0.68
	Asystole/bradycardia	20 (16%)	27 (12%)	0.33
	Sustained organized rhythm	38 (31%)	80 (37%)	0.26
**2**	VF	40 (32%)	65 (30%)	0.64
	Asystole/bradycardia	28 (23%)	42 (19%)	0.47
	Sustained organized rhythm	56 (45%)	111 (51%)	0.31
**3**	VF	28 (23%)	37 (17%)	0.23
	Asystole/bradycardia	34 (27%)	45 (21%)	0.18
	Sustained organized rhythm	62 (50%)	132 (62%)	0.04[a]
**4**	VF	16 (13%)	23 (11%)	0.56
	Asystole/bradycardia	40 (32%)	48 (23%)	0.05
	Sustained organized rhythm	69 (55%)	142 (67%)	0.03[b]
**5**	VF	10 (8%)	12 (6%)	0.36
	Asystole/bradycardia	42 (34%)	52 (24%)	0.05
	Sustained organized rhythm	70 (57%)	149 (70%)	0.02[c]
**>5**	VF	2 (2%)	0 (0%)	0.13
	Asystole/bradycardia	44 (36%)	56 (26%)	0.06
	Sustained organized rhythm	76 (62%)	157 (74%)	0.03[d]

*The data comparing fixed and escalating energy regimens were analyzed with chi-squired test.

a: Absolute difference, 12%; 95% CI 0.74 to 22.62.

b: Absolute difference, 12%; 95% CI 1.10 to 22.56.

c: Absolute difference, 13%; 95% CI 1.99 to 23.17.

d: Absolute difference, 15%; 95% CI 4.93 to 25.16.

© 2025 Tang H et al. *JAMA Network Open.*

eTable 2. Comparison of accumulative percentage of different resulted ECG rhythms between fixed-lower-energy and escalating-higher-energy defibrillations after each shock delivered

Number of Shock Delivered		Fixed-Lower-Energy Defibrillation n (%)	Escalating-Higher-Energy Defibrillation n (%)	p-value
1	VF	66 (53%)	111 (51%)	0.68
	Asystole/bradycardia	20 (16%)	27 (12%)	0.33
	Sustained organized rhythm	38 (31%)	80 (37%)	0.26
2	VF	106 (56%)	174 (53%)	0.57
	Asystole/bradycardia	28 (15%)	42 (13%)	0.54
	Sustained organized rhythm	56 (29%)	111 (34%)	0.30
3	VF	132 (58%)	209 (54%)	0.37
	Asystole/bradycardia	34 (15%)	45 (12%)	0.25
	Sustained organized rhythm	62 (27%)	132 (34%)	0.07
4	VF	148 (58%)	231 (55%)	0.45
	Asystole/bradycardia	40 (16%)	48 (11%)	0.11
	Sustained organized rhythm	69 (27%)	142 (34%)	0.05
5	VF	158 (59%)	241 (55%)	0.30
	Asystole/bradycardia	42 (16%)	52 (12%)	0.15
	Sustained organized rhythm	70 (26%)	149 (34%)	0.03[a]
>5	VF	182 (60%)	259 (54%)	0.08
	Asystole/bradycardia	44 (15%)	57 (12%)	0.27
	Sustained organized rhythm	76 (25%)	164 (34%)	0.008[b]

*The data comparing fixed and escalating energy regimens were analyzed with chi-squired test.

a: Absolute difference, 8%; 95% CI 0.80 to 22.62.

b: Absolute difference, 9%; 95% CI 2.36 to 15.64.

© 2025 Tang H et al. *JAMA Network Open.*

eTable 3 Comparison of percentage of different resulted ECG rhythms between fixed-lower-energy and escalating-higher-energy defibrillations for each shock delivered

Number of Shock Delivered		Fixed-Lower-Energy Defibrillation n (%)	Escalating-Higher-Energy Defibrillation n (%)	p-value
**1**	VF	66 (53%)	111 (51%)	0.68
	Asystole/bradycardia	20 (16%)	27 (12%)	0.33
	Sustained organized rhythm	38 (31%)	80 (37%)	0.26
**2**	VF	40 (61%)	65 (59%)	0.79
	Asystole/bradycardia	8 (12%)	15 (14%)	0.79
	Sustained organized rhythm	18 (27%)	31 (28%)	0.93
**3**	VF	28 (74%)	37 (63%)	0.28
	Asystole/bradycardia	4 (11%)	1 (2%)	0.08
	Sustained organized rhythm	6 (16%)	21 (36%)	0.04[a]
**4**	VF	16 (57%)	23 (66%)	0.29
	Asystole/bradycardia	6 (21%)	2 (6%)	0.12
	Sustained organized rhythm	6 (21%)	10 (29%)	0.57
**5**	VF	10 (71%)	12 (57%)	0.49
	Asystole/bradycardia	2 (14%)	2 (10%)	1.00
	Sustained organized rhythm	2 (14%)	7 (33%)	0.63
**>5**	VF	2 (20%)	0 (0%)	0.120
	Asystole/bradycardia	2 (20%)	4 (33%)	0.65
	Sustained organized rhythm	6 (60%)	8 (67%)	1.00

*The data comparing fixed and escalating energy regimens were analyzed with chi-squired test.

a: Absolute difference, 20%; 95% CI 1.53 to 38.08.

© 2025 Tang H et al. *JAMA Network Open.*

eTable 4. Comparison of accumulative percentage of different transient ECG rhythms between fixed-lower-energy and escalating-higher-energy defibrillations after each shock delivered

Number of Shock Delivered		Fixed-Lower-Energy Defibrillation n (%)	Escalating-Higher-Energy Defibrillation n (%)	p-value
**1**	VF	9 (7%)	13 (6%)	0.64
	Asystole/bradycardia	38 (31%)	67 (31%)	0.99
	Sustained organized rhythm	77 (62%)	137 (62%)	0.83
**2**	VF	16 (8%)	21 (6%)	0.79
	Asystole/bradycardia	60 (32%)	98 (30%)	0.40
	Sustained organized rhythm	114 (60%)	208 (64%)	0.70
**3**	VF	24 (11%)	26 (7%)	0.10
	Asystole/bradycardia	72 (32%)	112 (29%)	0.50
	Sustained organized rhythm	132 (58%)	248 (64%)	0.12
**4**	VF	32 (13%)	30 (7%)	0.02[a]
	Asystole/bradycardia	82 (32%)	123 (29%)	0.44
	Sustained organized rhythm	142 (55%)	268 (64%)	0.04[b]
**5**	VF	36 (13%)	30 (7%)	0.005[c]
	Asystole/bradycardia	86 (32%)	132 (30%)	0.58
	Sustained organized rhythm	148 (55%)	280 (63%)	0.02[d]
**>5**	VF	54 (18%)	32 (7%)	<0.001[e]
	Asystole/bradycardia	88 (29%)	142 (30%)	0.89
	Sustained organized rhythm	160 (53%)	306 (64%)	0.003[f]

*The data comparing fixed and escalating energy regimens were analyzed with chi-squired test.

a: Absolute difference, 5%; 95% CI 0.89 to 9.86.

b: Absolute difference, 8%; 95% CI 0.60 to 15.78.

c: Absolute difference, 7%; 95% CI 2.16 to 10.94.

d: Absolute difference, 8%; 95% CI 1.12 to 15.95.

e: Absolute difference, 11%; 95% CI 6.71 to 15.72.

f: Absolute difference, 11%; 95% CI 3.57 to 17.70.

© 2025 Tang H et al. *JAMA Network Open.*

eTable 5. Comparison of percentage of different transient ECG rhythms between fixed-lower-energy and escalating-higher-energy defibrillations for each shock delivered

**Number of Shock Delivered**		**Fixed-Lower-Energy Defibrillation n (%)**	**Escalating-Higher-Energy Defibrillation n (%)**	**p-value**
**1**	VF	9 (7%)	13 (6%)	0.64
	Asystole/bradycardia	38 (31%)	67 (31%)	0.99
	Sustained organized rhythm	77 (62%)	138 (63%)	0.82
**2**	VF	7 (11%)	8 (7%)	0.45
	Asystole/bradycardia	22 (33%)	31 (28%)	0.50
	Sustained organized rhythm	37 (56%)	70 (64%)	0.28
**3**	VF	8 (21%)	5 (8%)	0.08
	Asystole/bradycardia	12 (32%)	14 (24%)	0.39
	Sustained organized rhythm	18 (47%)	40 (68%)	0.12
**4**	VF	8 (29%)	4 (11%)	0.11
	Asystole/bradycardia	10 (36%)	11 (31%)	0.79
	Sustained organized rhythm	10 (36%)	20 (57%)	0.13
**5**	VF	4 (29%)	0 (0%)	0.02
	Asystole/bradycardia	4 (28%)	9 (43%)	0.49
	Sustained organized rhythm	6 (43%)	12 (57%)	0.50
**>5**	VF	2 (20%)	0 (0%)	0.20
	Asystole/bradycardia	2 (20%)	4 (33%)	0.65
	Sustained organized rhythm	6 (60%)	8 (67%)	1.00

© 2025 Tang H et al. *JAMA Network Open.*
