# Supplementary material for: Escalating vs Fixed Energy Defibrillation in Out-of-Hospital Cardiac Arrest Ventricular Fibrillation
Source: JAMA Netw Open. 2025 Apr 29;8(4):e257411. doi: 10.1001/jamanetworkopen.2025.7411 (PMC12042058; doi:10.1001/jamanetworkopen.2025.7411)
Supplement: Supplement 2. — Data Sharing Statement [file jamanetwopen-e257411-s002.pdf]

Data Sharing Statement

Tang. Escalating vs Fixed Energy Defibrillation in Out-of-Hospital Cardiac Arrest Ventricular Fibrillation. *JAMA Netw Open*. Published April 25, 2025.
doi:10.1001/jamanetworkopen.2025.7411

Data
Data available: No
